# Real-Time Monitoring of Underground Miners’ Status Based on Mine IoT System

**DOI:** 10.3390/s24030739

**Published:** 2024-01-23

**Authors:** Yufeng Jiang, Wei Chen, Xue Zhang, Xuejun Zhang, Guowei Yang

**Affiliations:** 1 CCTEG China Coal Research Institute, Beijing 100013, China; chenwei@ccrise.cn (W.C.); zhangxuejun@ccrise.cn (X.Z.); yangguowei@ccrise.cn (G.Y.); 2 Aerospace Information Research Institute, Chinese Academy of Sciences, Beijing 100190, China; zhangxue@aircas.ac.cn

**Keywords:** health monitoring, personnel positioning, wearable terminal, fatigue estimation, mine IoT

## Abstract

Real-time monitoring and timely risk warnings for the safety, health, and fatigue of underground miners are essential for establishing intelligent mines, enhancing the safety of production, and safeguarding the well-being of miners. This concerns the collection, transmission, and processing of relevant data. To minimize physical strain on miners, data collection functions are consolidated into two wearable terminals: an electronic bracelet equipped with reliable, low-power components for gathering vital sign data and transmitting them via Bluetooth and a miner lamp that integrates multi-gas detection, personnel positioning, and wireless communication capabilities. The gas sensors within the miner lamp undergo regular calibration to maintain accuracy, while the positioning tag supports round-trip polling to ensure a deviation of less than 0.3 m. Data transmission is facilitated through the co-deployment of 5G communication and UWB positioning base stations, with distributed MIMO networking to minimize frequent cell handovers and ensure a low latency of no more than 20 ms. In terms of data processing, a backpropagation mapping model was developed to estimate miners’ fatigue, leveraging the strong correlation between saliva pH and fatigue, with vital signs as the input layer and saliva pH as the output layer. Furthermore, a unified visualization platform was established to facilitate the management of all miners’ states and enable prompt emergency response. Through these optimizations, a monitoring system for underground miners’ status based on mine IoT technology can be constructed, meeting the requirements of practical operations.

## 1. Introduction

Underground mining operations involve various hazards [1,2], such as collapses, explosions, toxic substance exposure, and mechanical accidents, which pose serious risks to the safety and well-being [3] of underground miners. To enhance safety in production, it is crucial to establish an efficient perception system that can provide early warnings for safety hazards and health issues. This system should encompass data collection, transmission, and processing of information regarding each miner’s health status, fatigue level, exposure to environmental gases, and precise location.

In the medical field, evaluating human health often involves monitoring the four vital signs: respiration, body temperature, pulse rate, and blood pressure [4]. Recent advancements in wearable sensing devices [5] and Internet of Things (IoT) technology [6] have enabled remote monitoring [7] of vital signs, making it more convenient and accessible. This allows for the real-time capture of vital sign data from miners, enabling a prompt response to abnormal situations and the timely implementation of emergency measures [8,9]. However, current monitoring systems encounter specific limitations. Challenges include ensuring the daily wearability and maintenance of multiple sensors on different body parts. Furthermore, accurately and directly measuring fatigue levels [10] using wearable devices remains a challenge. While fatigue state assessment through measurements of heart rate [11], body movement [12], and skin conductivity [13] has been explored, it is often unreliable for evaluating miners’ conditions due to individual susceptibility to fatigue and external environmental factors.

In order to achieve timely risk warnings and emergency responses, it is essential to accurately measure environmental gas concentrations [14,15], such as methane (CH_4_), carbon monoxide (CO), and oxygen (O_2_), and to precisely track the location [16,17] of underground miners. While the relevant technology has matured, maintaining high accuracy while also ensuring a lightweight and portable solution remains a challenge.

Due to the unique environment, the deployment and application of wireless communication systems in mines face significant challenges. Safety concerns, as well as the presence of dust and combustible gases, impose limitations on the transmitting power of radio frequency signals, which in turn affects signal coverage [18]. Moreover, the operation of large-scale production equipment often introduces crosstalk in communication signals, further complicating signal processing [19]. Therefore, it is crucial to optimize wireless communication systems for underground scenarios to overcome these limitations and ensure reliable communication.

Once data reach the surface, it becomes crucial to establish a unified monitoring platform [20] that can process, manage, and display various status indicators, such as safety, health, and fatigue. This platform enables uniform state analysis and risk management and ensures seamless integration with the diverse systems operating within the mine.

## 2. Architecture of Monitoring System for Miner’s Status

**Figure 1:**
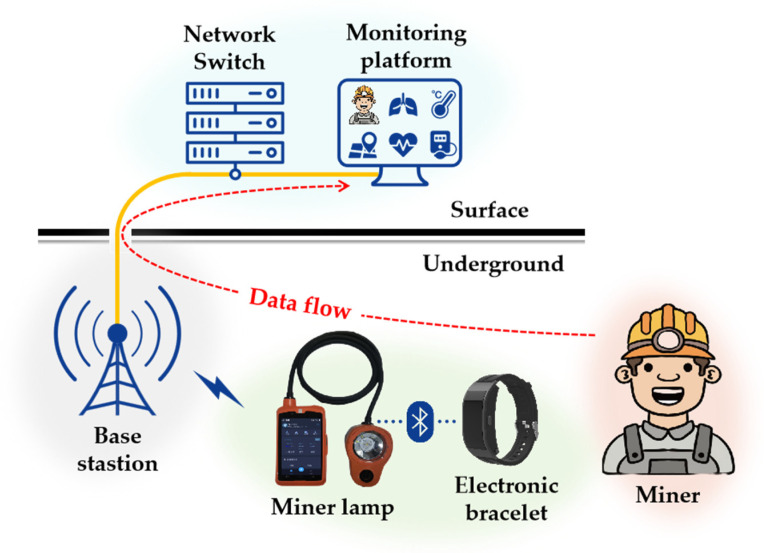
Monitoring system for miner’s status.

The monitoring system for the miner’s status, which is based on mine IoT technology, is illustrated in Figure 1. To strike a balance between reducing physical strain and ensuring monitoring capabilities, all underground miners are equipped with two highly integrated terminals: a lightweight electronic bracelet worn on the wrist and a waist-mounted smart miner lamp. The electronic bracelet collects vital signs such as respiration, body temperature, pulse rate, and blood pressure, synchronizing these data to the miner lamp via Bluetooth. The miner lamp is transformed from a basic lighting device to an intelligent terminal [21], featuring not only its primary purpose of providing underground illumination but also multi-gas detection, personnel positioning, and wireless communication. As mine intelligence advances, wireless communication systems such as 4G, 5G, and Wi-Fi have been extensively deployed in mines, providing efficient and reliable transmission channels for exchanging information between the underground and the surface. In the absence of Global Navigation Satellite Systems (GNSSs), spatial positioning intelligence in mines relies on technologies such as ZigBee or ultra-wideband (UWB). Therefore, the base station in the mine needs to provide both wireless communication and positioning coverage, usually using a combination of technologies such as 4G and ZigBee, 5G and UWB, or other suitable options. Lastly, the data of all miners converge from underground to the surface and undergo data processing procedures, such as health and safety assessment and fatigue estimation, before being uniformly presented on the monitoring platform.

Taking the system deployed in a coal mine located in Northwest China as an example, the main parameters are shown in Table 1.

## 3. Realization Methods and Results

### 3.1. Vital Signs Collection and Synchronization

In order to ensure the accurate capture of miners’ vital signs and enable real-time reporting, the development of a customized electronic bracelet design must adhere to several key principles. Firstly, non-invasive measurements [22] should be utilized to avoid any potential environmental interference [23]. Secondly, the design should prioritize high integration and lightweight construction to minimize additional burden on the miners. Lastly, strict adherence to safety and explosion-proof requirements in the mine is essential.

**Figure 2:**
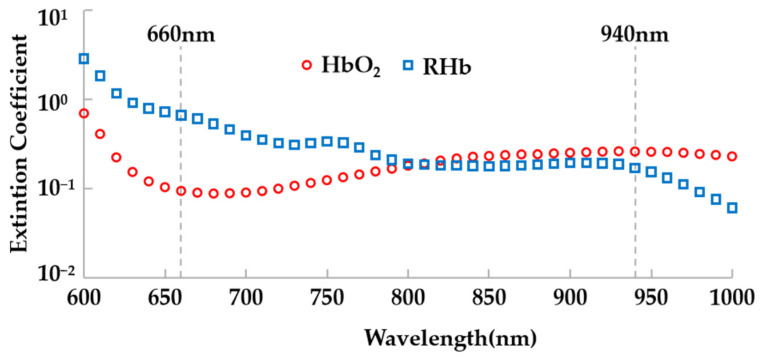
Absorption spectra of human tissue for HbO_2_ and RHb.

Blood oxygen saturation is a crucial physiological parameter in the field of respiration and circulation, representing the proportion of oxygen-saturated hemoglobin in the blood compared to the total hemoglobin [24]. In clinical practice, the widely used pulse oximeter (SpO_2_) [25] is employed to measure blood oxygen saturation, calculated using the following formula:

SpO_2_ = HbO_2_/(HbO_2_ + RHb) (1)

where HbO_2_ refers to oxyhemoglobin, while RHb represents total hemoglobin. The calculation of SpO_2_ relies on the different absorption rates of oxyhemoglobin and total hemoglobin for specific wavelengths of light. Typically, the reflectance of human tissue across two distinct wavelengths, such as 660 nm and 940 nm, is analyzed to determine SpO_2_. Figure 2 provides an illustration of the light absorption by different components of the blood.

Body temperature measurement can be achieved using a pyroelectric infrared detector [26], which detects infrared radiation using pyroelectric properties in specific materials and enables accurate body temperature measurement. This method is reliable and not affected by factors such as sweat or ambient temperature.

Another widely used technique for health monitoring is photoplethysmography (PPG), which utilizes changes in light reflection from human tissue to extract pulse waves [27]. By analyzing characteristics of the pulse wave signal, such as its frequency, and establishing a regression equation for blood pressure estimation based on pulse wave data, real-time blood pressure measurement can be achieved [28]. The electronic bracelet that uses PPG to measure blood pressure shows readings that deviate within ±3 mmHg compared to the calibrated mercury sphygmomanometer, as shown in Table 2.

Due to the high real-time requirements but low data rate, data synchronization between the electronic bracelet and the miner lamp can be achieved through the Bluetooth protocol. To achieve a lightweight design, the electronic bracelet utilizes a Bluetooth Low Energy (BLE) module to reduce power consumption by using smaller packets and spaced connections [29]. Similar to traditional Bluetooth technology, BLE can also handle interference through adaptive frequency hopping (AFH) [30]. It supports equiprobable hops within 37 data channels, usually employing the mode 37 method, which can be described by the following equation:

f_n+1_ = (f_n_ + X) mod 37 (2)

where f_n_ and f_n+1_ represent the channel index before and after frequency hopping, X is the frequency hopping parameter, an integer ranging from 5 to 16, and mod is the remainder operator.

By integrating highly reliable and low-power components, the electronic bracelet is capable of collecting four vital signs and reporting them to the miner lamp in real time. Moreover, the electronic bracelet is powered by a LiMn_2_O_4_ (lithium manganese oxide) battery [31], meeting the explosion-proof requirements of coal mines.

### 3.2. Multi-Gas Detection and Personnel Positioning

**Figure 3:**
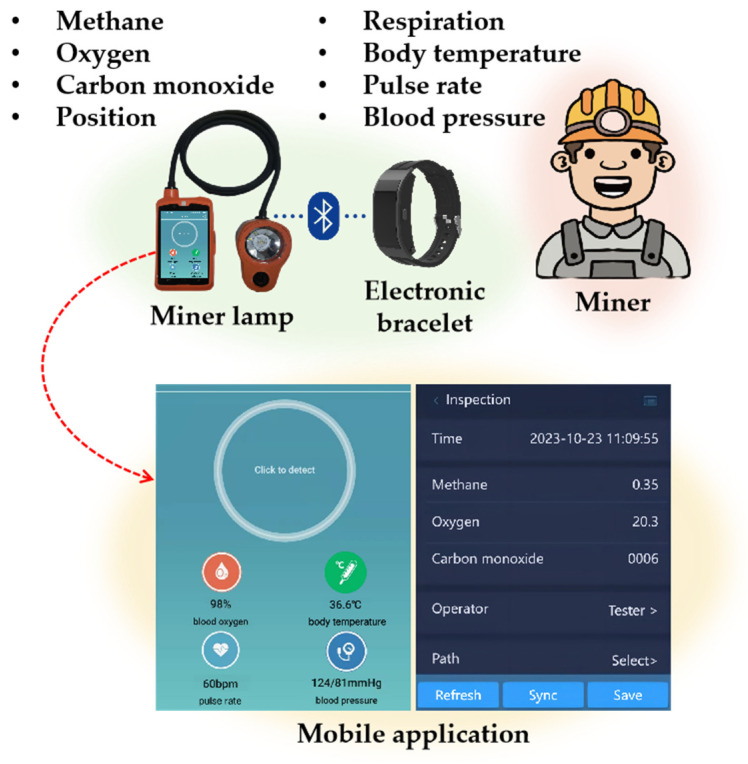
Health and safety data collection based on wearable terminals.

The modern miner lamp has undergone significant evolution, now functioning as a highly integrated terminal that combines the features of a smartphone, a multi-gas detector, and a positioning tag. Alongside the electronic bracelet, the miner lamp forms a comprehensive health and safety data acquisition system aimed at ensuring the well-being of underground miners, as illustrated in Figure 3.

Although tunable diode laser absorption spectroscopy (TDLAS) [32,33] offers outstanding accuracy and precision for multi-gas detection, it presents challenges in terms of miniaturization. As a solution, traditional yet compact electrochemical sensors are employed in miner lamps to detect gases such as CH_4_, CO, and O_2_, with each gas requiring a dedicated sensor. The output voltage of electrochemical sensors generally demonstrates a linear relationship with gas concentration, as represented by the following equation:

U = k × C(3)

where U, k, and C denote the output voltage, linear slope, and gas concentration, respectively.

**Figure 4:**
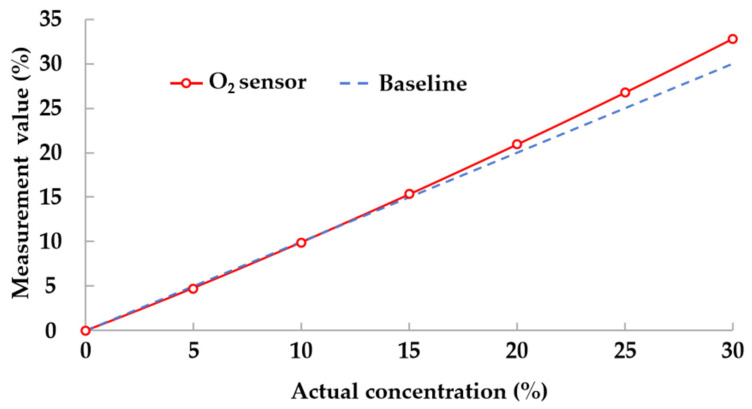
Measurement deviation of O_2_ sensor in original state.

In practice, relying solely on formula (3) may not yield accurate measurements due to the slight nonlinearity in the sensor. For example, Figure 4 illustrates the measurement deviation of the O_2_ sensor in its original state. Our essential task is to achieve precise measurements by establishing a mapping relationship between the measured values and the actual gas concentration through proactive calibration. Given the potential changes in the properties of electrochemical sensors over time, regularly calibrating the gas detection function in the miner lamp is essential to maintain accuracy.

**Figure 5:**
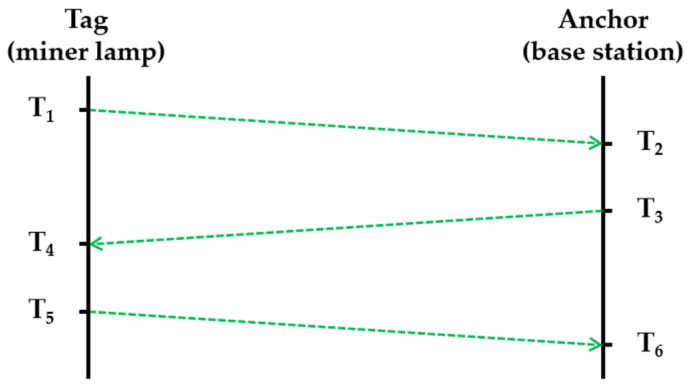
Schematic diagram of the DS-TWR method.

UWB is widely acknowledged as the optimal choice for personnel positioning in mines [34,35], due to its impressive range, precision, and robustness in challenging tunnel environments. The theoretical accuracy of UWB ranging can achieve millimeter-scale or even higher resolutions due to the picosecond-level timestamp accuracy of pulses. However, many factors, including clock offset between the anchor and tag, can significantly impact actual positioning accuracy. To address this issue, the double-sided two-way ranging (DS-TWR) method is adopted [36], achieved through round-trip exchanges between the anchor (base station) and the tag (miner lamp) via polling, as illustrated in Figure 5. The ranging process begins with the tag initiating a signal transmission along with a timestamp, and the signal undergoes 3-trip exchanges between the tag and anchor via polling. During the final trip, an end-of-transmission signal is added to indicate the completion of a full ranging.

By analyzing the time differences of these exchanges, represented by T_1_ to T_6_, the distance value is computed using the following equation that establishes a relationship between speed and time:

d = [(T_4_ − T_1_ − T_32_) + (T_6_ − T_3_ − T_54_)] × c/4 (4)

where T_32_ equals T_3_ − T_2_, and T_54_ equals T_5_ − T_4_. The symbol c represents the velocity of the electromagnetic wave, which is equal to 299,792,458 m/s. Personnel positioning can be achieved by obtaining the distance between the miner lamp and the two nearby base stations through DS-TWR, ensuring that the UWB positioning error does not exceed 0.3 m, as shown in Table 3.

### 3.3. Base Station Deployment and Networking

**Figure 6:**
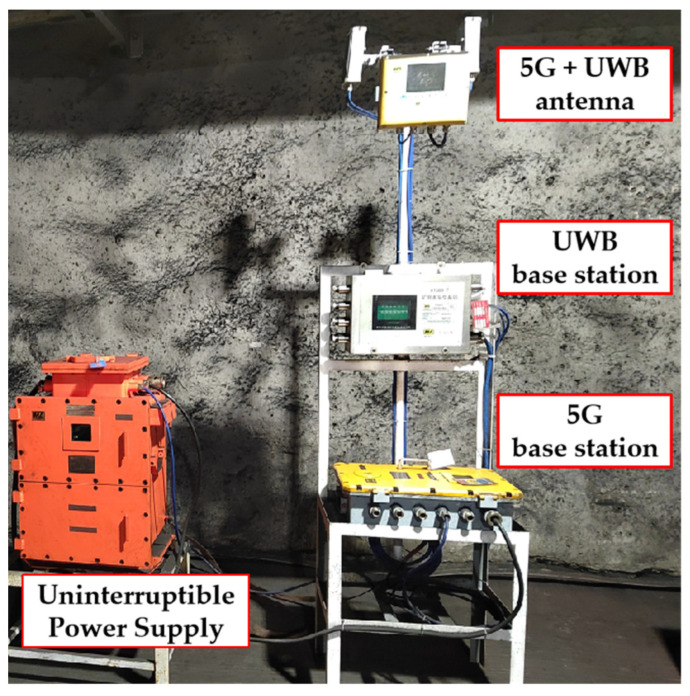
Co-deployment of 5G and UWB base stations.

In practical mine deployment, the positioning base station usually relies on the communication base station for data transmission and typically follows a co-deployment approach, as depicted in Figure 6. By utilizing the 5G and UWB combination, the coverage range of the positioning base station is 2 to 3 times that of the communication base station. Consequently, the positioning base station is deployed at intervals from the communication base station.

**Figure 7:**
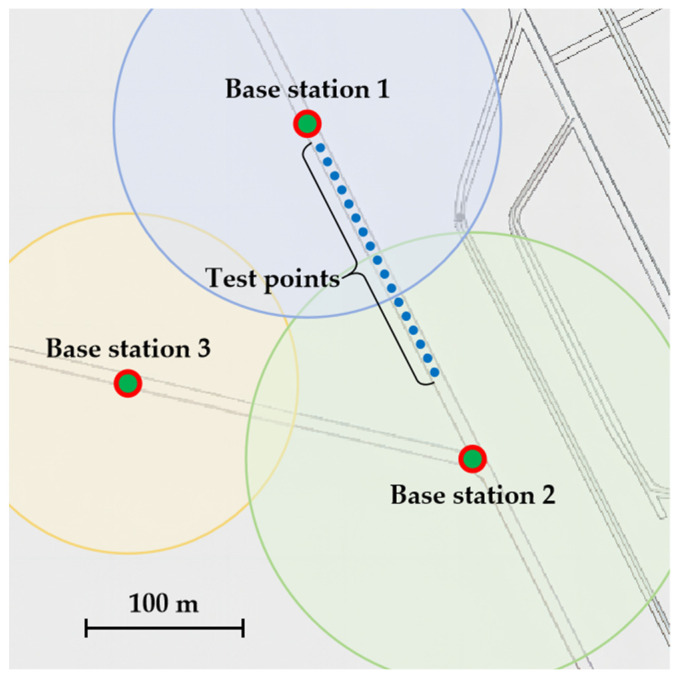
Schematic diagram of 5G base station coverage in the mine.

When considering the deployment of 5G base stations operating on the 3.5 GHz frequency band, it is worth noting that the coverage radius of a single base station is approximately 200 m [37]. Signal quality rapidly declines beyond this range, as illustrated in Figure 7, which shows a partial map of the base station coverage in the mine. In the traditional cellular network architecture [38], each base station represents a cell, leading to frequent cell handovers [39] when user terminals are on the move, compromising communication performance, particularly in terms of latency. Furthermore, the complex electromagnetic environment and multi-path effects in confined spaces further limit the performance of individual base stations.

**Figure 8:**
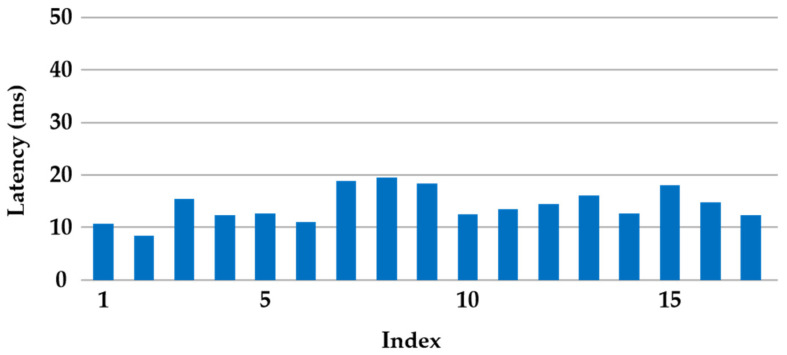
The results of latency at different locations within the tunnel.

To address these challenges, distributed multiple-input multiple-output (D-MIMO) [40,41] networking is implemented. This technique effectively enables collaboration among neighboring base stations, allowing them to collectively serve a single user terminal and thereby enhancing overall network coverage. Additionally, as a form of MIMO technology, it naturally exhibits characteristics such as interference suppression and fading suppression. Within a mine tunnel, specific test points between two neighboring 5G base stations, as marked in Figure 7, were selected to measure the latency changes from underground terminals to the surface server during continuous movement, as depicted in Figure 8. Upon repeated verification, it was observed that the maximum latency does not exceed 20 ms, indicating the transformation of this tunnel into a cell-free area where changes in position have no impact on latency.

### 3.4. Fatigue Level Estimation

Following prolonged, high-intensity labor, the body generates an excess of lactic acid [42,43], resulting in reduced pH levels of bodily fluids including blood, sweat, and saliva [44,45]. While real-time non-invasive monitoring of any kind of bodily fluids may be impractical, establishing a correlation between vital signs and salivary pH in advance allows for the estimation of fatigue levels using data from electronic bracelets.

**Figure 9:**
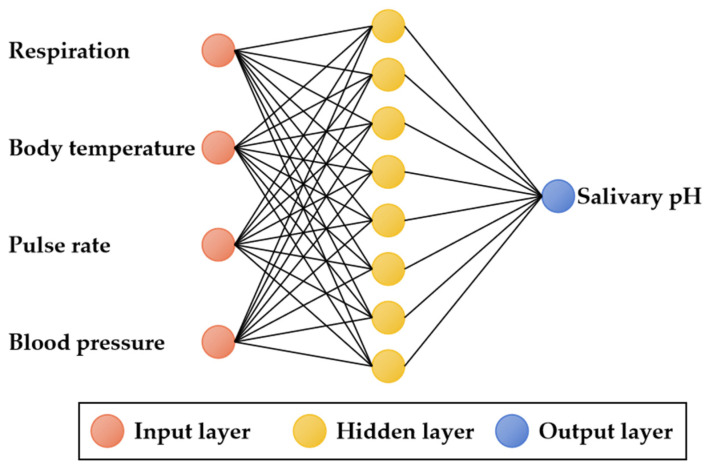
Structure diagram of 3-layer neural network.

Backpropagation [46] is a mature neural network algorithm that learns through data training to achieve the mapping of input and output. In this application, we use a 3-layer neural network with appropriate parameter settings to simulate complex nonlinear mappings. The input layer consists of four neurons: respiration, body temperature, pulse rate, and blood pressure. These inputs are processed through the hidden layer and mapped to the output layer, which represents the pH value of saliva. The number of neurons in the hidden layer can be determined using the following empirical formula:

m = sqrt (n + l) + a(5)

where m represents the number of neurons in the hidden layers, n is the number of neurons in the input layer, and l is the number of neurons in the output layer. The constant a ranges from 1 to 10. In the context of the current scenario, selecting a value of 6 for a strikes a more optimal balance between achieving high accuracy and maintaining low complexity. As a result, we have set m to 8, which leads to the establishment of the neural network structure as shown in Figure 9.

In a production mine, operations are typically carried out in three shifts throughout the day. At the end of each shift, saliva samples are collected and tested. Salivary pH is influenced by various physiological activities within the human body, including fatigue, but not limited to it. Therefore, our focus is not on the absolute value of pH but on the changes caused by the fatigue level.

∆pH = pH_0_ − pH_shift_ − ∆pH_id_ (6)

where ∆pH represents the relative change in pH, pH_0_ represents the measured value of pH, pH_shift_ represents the average pH value for each shift, and ∆pH_id_ is the average ∆pH for each individual miner in the absence of fatigue. 

**Figure 10:**
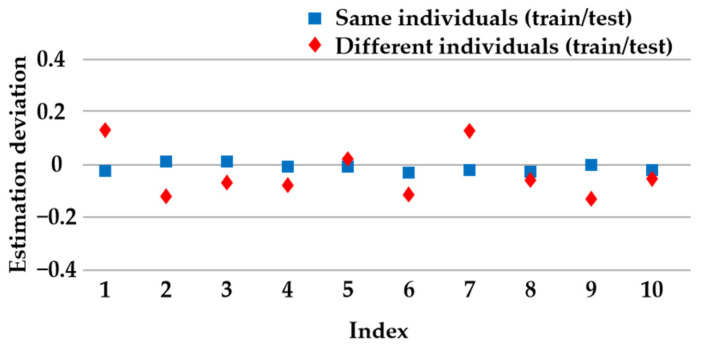
Estimation deviation of pH values using neural network.

The neural network is continuously trained to update the weights and thresholds using datasets derived from the same individuals for both training and testing. This approach ensures a pH value judgment error that does not exceed 0.05, as shown in Figure 10, even when considering different individuals, thus remaining within an acceptable threshold of 0.2 for the purpose of fatigue estimation.

### 3.5. Data Management and Emergency Response

In general, data management is composed of two software components. The first is a mobile application installed in the miner lamp. Its primary functions are to display data on the screen and upload it to the server. The second component is a monitoring platform located on the surface server. This platform assesses the safety and health status of all miners, estimates their fatigue levels, and presents the results via a web interface as shown in Figure 10. Additionally, it receives information from the server through MQTT (Message Queuing Telemetry Transport) and forwards it to the electronic bracelet.

**Figure 11:**
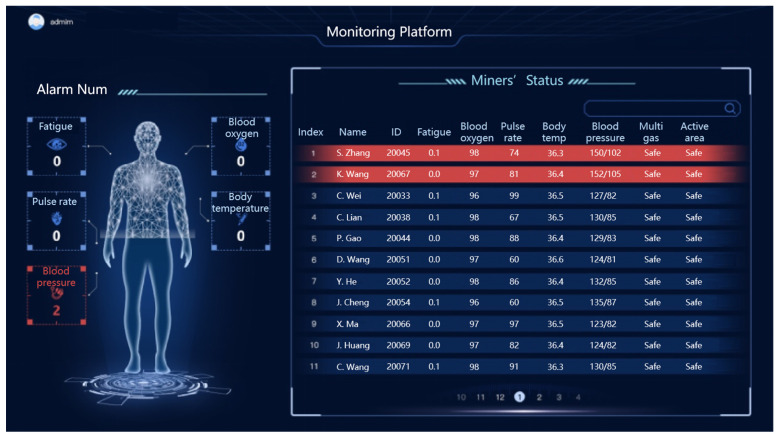
Interface of monitoring platform for miners’ status.

**Figure 12:**
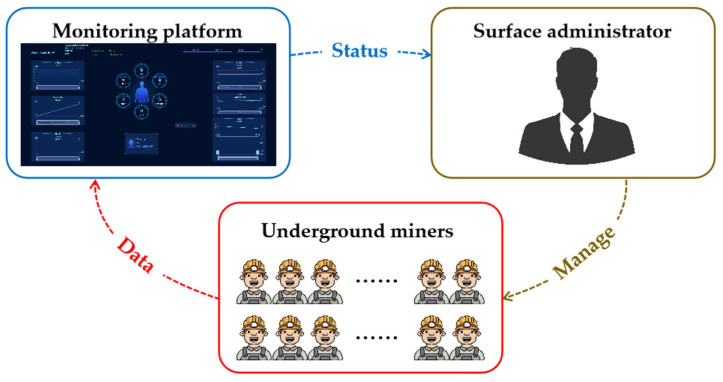
Interaction among underground miners, monitoring platform, and surface administrator.

Figure 11 depicts the interaction among underground miners, the monitoring platform, and the surface administrator, who is able to promptly respond to any anomalies. The entire cycle enables early warnings of safety hazards and health issues, providing assurance of underground emergency response. The interaction among underground miners, monitoring platform, and surface administrator is shown in Figure 12.

Furthermore, the monitoring platform can be seamlessly interconnected with the mine’s broadcasting, scheduling, video, and other systems, enabling real-time linkage to collectively enhance the safety and productivity of the mine.

## 4. Conclusions

In this paper, we propose a real-time monitoring system based on mine IoT technology to assess the safety and health, as well as estimate the fatigue level, of underground miners. We discuss this system from several aspects, including data collection, transmission, and processing. Unlike conventional solutions, all components for data acquisition are integrated into a lightweight electronic bracelet and a smart mine lamp, with functions optimized for vital signs collection, multi-gas detection, and personnel positioning to meet specific requirements. Through the co-deployment of base stations and the implementation of a distributed MIMO network, effective coverage of communication and positioning signals can be achieved, overcoming the limitations of cellular networks. To address the challenge of directly measuring fatigue levels, we constructed a mapping model based on vital signs and saliva pH value to estimate fatigue. By conducting a comprehensive state analysis and implementing risk management, alerts regarding safety hazards and health issues can be generated from a visualization platform on the surface.

The proposed real-time monitoring system, which utilizes IoT technology, has been successfully operational in a coal mine in Northwest China for over six months. It offers an effective way to monitor the well-being of all miners, thereby minimizing the risk of accidents and enhancing safety throughout the production process. This system stands out from its predecessors due to its ability to assess fatigue levels and to perform integrated processing of multi-dimensional data, all without imposing additional burdens on the miners. According to initial user feedback, there have been no safety incidents related to the physical condition of miners since the system was implemented.

Looking ahead, plans are in place to extend this advanced monitoring system to other mines via renovation. For example, in open-pit mines, the GNSS will replace UWB for personnel positioning. Moreover, sophisticated data protection methods, like blockchain technology, will be adopted to strengthen the security and confidentiality of the data.

## 5. Patents

There are three Chinese patents resulting from the work reported in this paper: ZL202010454491.2, ZL202211346766.6, and ZL202310627721.4, corresponding to Section 3.2, Section 3.3, and Section 3.4, respectively.

## Figures and Tables

**Table 1:** Parameters of the monitoring system for miner’s status.

Items	Parameters
Underground miner	- Three shifts per day, with a maximum of 400 miners per shift
Electronic bracelet	- Collection of vital signs: respiration, body temperature, pulse rate, blood pressure
	- Data synchronization: Bluetooth
Miner lamp	- Wireless communication: 5G, Wi-Fi
	- Gas detection: methane, oxygen, carbon monoxide
	- Personnel positioning: UWB
Base station	- 5G: Upload ≥ 450 Mbps, delay ≤ 20 ms
	- UWB: error ≤ 30 cm
Monitoring platform	- Assessment: health and safety
	- Estimation: fatigue level
	- Maximum concurrent number: up to 5000 users

**Table 2:** Comparison of blood pressure test results.

Test Time	Electronic Bracelet		Sphygmomanometer	
	Systolic	Diastolic	Systolic	Diastolic
8:00	126 mmHg	83 mmHg	125 mmHg	84 mmHg
10:00	132 mmHg	87 mmHg	129 mmHg	85 mmHg
12:00	135 mmHg	88 mmHg	133 mmHg	86 mmHg
14:00	128 mmHg	83 mmHg	128 mmHg	82 mmHg
16:00	122 mmHg	82 mmHg	120 mmHg	81 mmHg

**Table 3:** Positioning results based on the DS-TWR.

	Absolute Distance			
	1.00 m	125.00 m	250.00 m	400.00 m
Test 1	1.09 m	125.03 m	250.05 m	400.07 m
Test 2	1.07 m	125.02 m	250.05 m	400.04 m
Test 3	1.12 m	125.03 m	250.12 m	399.99 m
Test 4	1.17 m	125.04 m	250.06 m	400.06 m
Test 5	1.12 m	125.02 m	250.10 m	400.07 m
Test 6	1.12 m	125.05 m	250.09 m	400.12 m
Test 7	1.04 m	124.98 m	250.11 m	400.03 m
Test 8	1.06 m	125.10 m	250.02 m	400.07 m
Test 9	1.16 m	125.00 m	250.05 m	400.05 m
Test 10	1.11 m	124.99 m	250.11 m	400.07 m

## Data Availability

Dataset available on request from the authors.
